# A Review on Green Synthesis of TiO_2_ NPs: Photocatalysis and Antimicrobial Applications

**DOI:** 10.3390/polym14071444

**Published:** 2022-04-01

**Authors:** Vishal Verma, Mawaheb Al-Dossari, Jagpreet Singh, Mohit Rawat, Mohamed G. M. Kordy, Mohamed Shaban

**Affiliations:** 1 Department of Nanotechnology, Sri Guru Granth Sahib World University, Fatehgarh Sahib 140406, India; vishuu210@gmail.com (V.V.); mohitnano.nit@gmail.com (M.R.); 2 Department of Physics, Dhahran Aljanoub, King Khalid University, Abha 61421, Saudi Arabia; mdosri@kku.edu.sa; 3 Research Center for Advanced Materials Science (RCAMS), King Khalid University, Abha 61413, Saudi Arabia; 4 Department of Chemical Engineering, Chandigarh University, Gharuan, Mohali 140413, India; 5 Centre for Research and Development, Chandigarh University, Gharuan, Mohali 140413, India; 6 Nanophotonics and Applications (NPA) Lab, Physics Department, Faculty of Science, Beni-Suef University, Beni-Suef 62514, Egypt; m.kordybio@science.bsu.edu.eg (M.G.M.K.); mssfadel@aucegypt.edu (M.S.); 7 Biochemistry Department, Faculty of Science, Beni-Suef University, Beni-Suef 62521, Egypt; 8 Department of Physics, Faculty of Science, Islamic University of Madinah, Al-Madinah Al-Munawarah 42351, Saudi Arabia

**Keywords:** green synthesis, plants, TiO_2_ NPs, photocatalysis, dyes photodegradation, antimicrobial activity

## Abstract

Nanotechnology is a fast-expanding area with a wide range of applications in science, engineering, health, pharmacy, and other fields. Nanoparticles (NPs) are frequently prepared via a variety of physical and chemical processes. Simpler, sustainable, and cost-effective green synthesis technologies have recently been developed. The synthesis of titanium dioxide nanoparticles (TiO_2_ NPs) in a green/sustainable manner has gotten a lot of interest in the previous quarter. Bioactive components present in organisms such as plants and bacteria facilitate the bio-reduction and capping processes. The biogenic synthesis of TiO_2_ NPs, as well as the different synthesis methods and mechanistic perspectives, are discussed in this review. A range of natural reducing agents including proteins, enzymes, phytochemicals, and others, are involved in the synthesis of TiO_2_ NPs. The physics of antibacterial and photocatalysis applications were also thoroughly discussed. Finally, we provide an overview of current research and future concerns in biologically mediated TiO_2_ nanostructures-based feasible platforms for industrial applications.

## 1. Introduction

Nanotechnology deals with atoms and molecules at the supermolecule scale [1,2]. Due to the escalating surface area to volume, there is a drastic change in the physicochemical characteristics of nanomaterials at this level [3]. Along with its size, structure, and physicochemical and biological characteristics, nanotechnology has a diverse set of applications in a multitude of fields such as industries, notably mechanical, electronic, imaging specific targeting, and molecular diagnosis [4]. Nanoparticles (NPs) are being used in more and more purposes every day, covering medical, cosmetology, pharmaceuticals, and power. Organic and inorganic NPs are the two basic types of NPs. Micelles, liposomes, chitosan, ferritin, dendrimers, and other organic NPs are examples. Inorganic NPs are divided into three groups: metal nanoparticles; semiconductor NPs; and magnetic NPs [5]. 

Due to their intriguing thermal, optical, electrical, and magnetic characteristics, metal oxide nanoparticles, particularly TiO_2_ nanoparticles, are widely employed. Titania is the only titanium oxide that occurs naturally [6,7,8,9]. TiO_2_ is an odorless, brilliantly white powder that, under normal conditions, is hydrophobic in nature. It is a highly stable material that also works well as an opacifier. As a result of its key properties, like minimal cost, great oxidizing strength, high chemical stability, high refractive index, and the existence of oxygen-containing functional groups in its lattice, TiO_2_ NPs are largely employed as a semiconductor material. In 2011, global TiO_2_ output surpassed 10,000 tons per year [10]. They can also be employed to biodegrade a variety of microorganisms, including bacteria, viruses, and cancer cells. UV light resistant oxides, toothpastes, papers, food colorants, paints, plastics, and inks all contain them. TiO_2_ NPs are the most efficient solar collectors, absorbing 3–4% of solar energy. As a result, they are well-known photocatalysts for hydrogen production, as well as for the degradation of hazardous chemical compounds in water [11]. Surface properties and topologies of TiO_2_ NPs are distinct. TiO_2_ is a whitish metal oxide that is a solid inert compound. Anatase, rutile, and brookite are the three distinct polymorphs found in TiO_2_ NPs. Anatase and rutile have similar qualities (such as gloss, rigidity, and densities) and geometric symmetry (tetragonal) [12]. TiO_2_ is an insoluble, fire-resistant, high thermal stability metal oxide that is not categorized as dangerous. The atomic number of titanium in TiO_2_ is 22 from the IV B group, whereas the atomic number of oxygen is eight from the VI A group [1]. It also has good characteristics including hydrophobic nature and a wide bandgap. Dye-sensitized solar cells, self-cleaning, photocatalysis, charge-spreading devices, chemical sensors, microelectronics, electrochemistry, antimicrobial products, and textiles are all examples of industrial applications [13]. Degradation of harmful compounds is based on the catalytic oxidation of hydrocarbons [14,15,16,17,18]. TiO_2_ NPs are likely the most significant scientific interest across all metal oxides in photocatalytic, antimicrobial, and antibacterial effective applications due to their superior properties [19,20,21]. The use of nano-sized TiO_2_ in photocatalytic wastewater treatment is a very successful method for decomposing and eliminating resistant organic and inorganic contaminants in wastewater [22,23,24].

Chemical vapor deposition (CVD), electrochemical deposition, sol-gel technique, hydrothermal crystallization, and chemical precipitation are the most common ways of making TiO_2_ NPs. [25]. All of the processes listed above are time and money-intensive, and they all require high temperatures, pressures, and harmful chemicals to complete, limiting their manufacturing and potential medicinal applications [26]. Consequently, green synthesis is a frequently used process for the production of NPs. Green synthesis is a naturally adaptable, environmentally sound, and cost-effective technique for large–scale NP synthesis [27,28]. Plant extracts operate as reducing agents, and the same reducing agent can be employed to make a variety of metallic nanoparticles [29,30,31,32,33]. Plant extracts that are used in the synthesis of NPs can be leaves, roots, fruits, seeds, or beans [27,34,35,36,37]. Green TiO_2_ nanoparticles are prepared using several extracts for multifunctional applications. Plant-based nanoparticles could be valuable in a variety of industries, including medicinal, food, catalysis, and cosmetics. According to previous findings, green sources are always utilized as a stabilizer and reducing agent in the production of NPs with structured shape and size.

**Figure 1:**
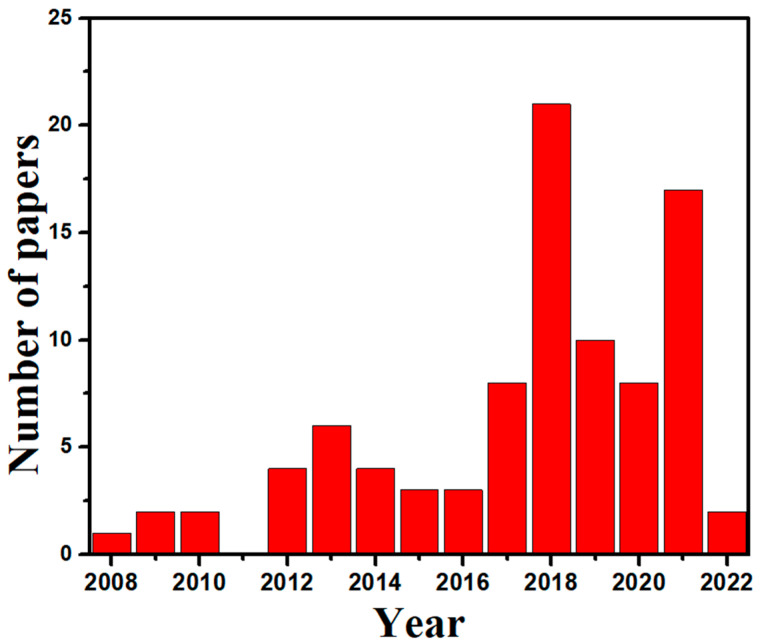
Histogram shows the proportion of papers published on green techniques for TiO_2_ NPs.

The current review focuses on plant and microorganism-based green synthesis of TiO_2_ NPs, including detailed methods and practical applications. To begin, the green synthesis of numerous biological extracts has been thoroughly addressed. Second, using an in-depth characterization investigation on green synthesized TiO_2_ NPs, a comprehensive examination of the morphological and structural characteristics of NPs is explored. Finally, the benefits of green synthesis, particularly for photocatalysis and antimicrobial applications, are also discussed. Finally, the conclusion and future outlook have been discussed. We also gathered paper publishing data from PubMed (Figure 1), which shows that academics are increasingly interested in green synthesis of TiO_2_ NPs.

## 2. Synthesis of TiO_2_ NPs by Different Methods

**Figure 2:**
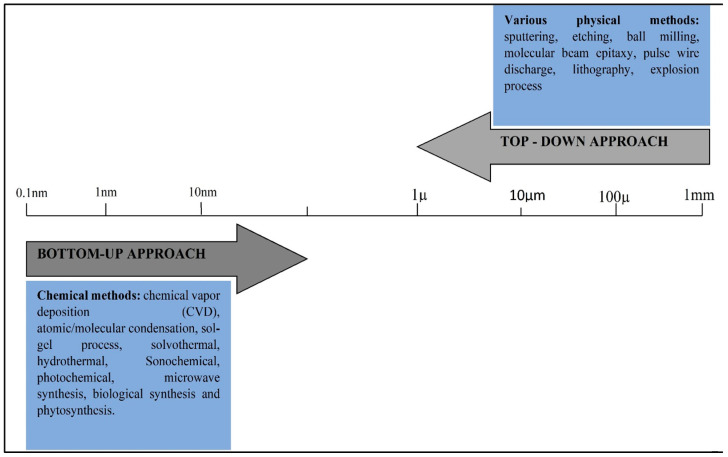
Nanoparticle synthesis methods.

The two primary methodologies for the synthesis of nanomaterials are top-down and bottom-up approaches as shown in Figure 2.

- Top-down: size reduction from bulk materials
- Bottom-up: material synthesis from the atomic level

### 2.1. Top-Down Approach

Bulk material is turned into a nano product using a top-down technique. For size reduction, both physical and chemical approaches were applied. Sputtering, pulse wire discharge, physical milling/ball milling, etching, evaporation–condensation reaction, pulse laser ablation, and lithography are some of the processes employed in the top-down approach. However, there are certain disadvantages to the top-down approach, the most significant of which is that defects are imposed on the product’s surface. This could affect the product’s surface properties and other physical characteristics [38].

### 2.2. Bottom-Up Approach

The materials were built up from the bottom in the bottom-up approach: atom by atom, molecule by molecule, and cluster by cluster. Most nanostructures with the potential to make a homogeneity, size, and morphology are synthesized using this process. Chemical synthesis is offering a broad range of techniques like chemical vapor deposition, solvothermal, polymer condensation, sol-gel method, aerosol methodology, electrochemistry, pyrolysis, thermal decomposition, frameworks, plasma, and spinning also available Green synthesis, in particular, controlling the process in the bottom-up synthesis to decrease particle development. As a result, scientists can state that the bottom-up technique is crucial in the creation of nanostructures and nanomaterials [39,40,41]. Almost all of these nanomaterial synthesis methods are employed, however, if we consider that, the bottom-up approach is the most efficient as it is beneficial and achieves perfection at the atomic scale. The bottom-up technique is also used since the green synthesis routes have been thought-out to be a practical strategy due to the employment of non–toxic, cost-effective, and ecologically friendly matter [42,43]. Natural various plant extracts are employed in green synthesis. In green chemistry, the plant extract serves as a capping and reducing agent, and it is blended with a simple precursor salt [44]. The plant extract’s phytochemicals can then reduce and stabilize the nanomaterials. With the new revolution, a lot of work has been done in green synthesis to synthesize a variety of metal NPs such as Cu, Pt, Pb, Ag, Au, Zn, and so on [45]. Phyto-synthesis of TiO_2_ NPs utilizing various plant extracts is discussed in this review. In this regard, recent research has been compiled from the literature to summarize research efforts [45].

### 2.3. Green Synthesis

Green synthesis is considered to play a key role in the current engineering and science field. As a result of their distinctive properties of biosynthesized nanomaterials, which are used for the treatment of water and contaminated sites [45]. Nanoparticles are of keen interest due to their special attributes, such as their exceedingly small size, high surface area to volume ratio, surface modifiability, and size-dependent properties [14,27]. These nanoparticles also showed their applications in the medical field and pharmacy [38]. Nowadays, vast research is being conducted on the biological system. The biological synthesis of nanomaterials used bacteria, fungi, yeast, and plants. Due to their cost-effectiveness, these synthesis approaches have been the subject of widespread interest. The biologically synthesized nanoparticles have a wide range of applications in the field of contaminant remediation, as well as antibacterial, antifungal, high catalytic, and photochemical activity [45]. Au and Ag NPs are two of the most widely produced NPs, with numerous biomedical applications. The photocatalytic activity of Au and Ag nanoparticles was good. Nanotechnology and biotechnology, which deal with microorganisms like bacteria, fungi, yeasts, algae, and plants, are the most promising fields of research. The use of microorganisms to synthesize nanoparticles revealed a prospective mechanism. The inorganic nanomaterials were produced with the help of the above-mentioned living organisms, and they showed great results. Solubility plays an important role in the resistance, which is caused by the bacterial cell for reactive ions [46,47]. The rate of synthesis of NPs with microbes is very slow and there are limited methods, by which NPs are fabricated with desirable shape and size. The nanomaterials that are routed by plants are cost-effective and very simple methods. In these methods, there is no need for high temperature and toxic chemicals, or high pressure. As a result, these methods are environmentally friendly. Today’s focus was on green synthesis, and with the help of plants, the NPs were very stable and in the proper form and size. Another benefit of green synthesis is that the chance of contamination is quite minimal. The plants contain many phytochemicals, which help in the production of nanomaterials and NPs. Plants provide a variety of phytochemicals that are commonly utilized and inexpensive in the synthesis of nanomaterials and nanoparticles. The phytochemicals also play an important role as they help at the time of photocatalytic activity applications. They help in the oxidation and reduction reactions at the photocatalytic activity time of the organic dyes.

### 2.4. Plant-Based synthesis of Titanium Dioxide NPs

**Figure 3:**
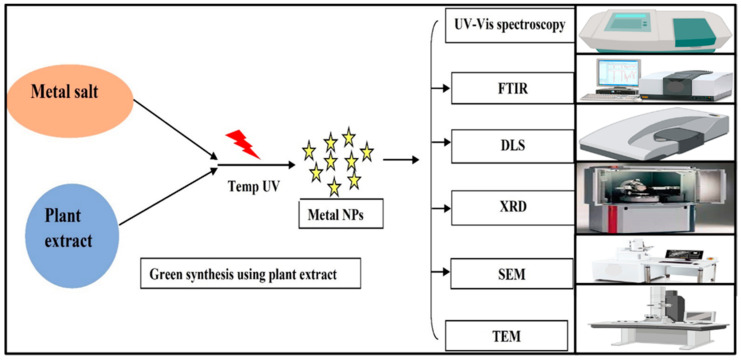
Schematic diagram of the preparation process of nanoparticles via plant extract.

The green synthesis studies have been achieved on extracts of leaves as plant extract contains a rich source of metabolites. Figure 3 shows schematic diagram of the preparation process of nanoparticles via plant extract. Kashale et al. used *Cicer arietinum* L. extract to mediate TiO_2_ NPs in 2016 using TiCl_4_ (titanium tetrachloride) as a precursor [48]. They have reported that the prepared biosynthesized TiO_2_ (Bio–TiO_2_) NPs is a worthy way for the rapid synthesis of NPs. The morphology of Bio–TiO_2_ NPs showed a crystal structure and other properties were investigated by Raman spectroscopy, X-ray diffraction (XRD), thermogravimetric analysis (TGA), transmission electron microscopy (TEM), and BET surface area measurement system. Rao et al. in 2015 obtained the TiO_2_ NPs by employing the leaf extract of Aloe Vera. Aloe Vera plant is the oldest herbal medicinal plant that contains mineral amino acids and fatty acids and high vitamins. It is also used for skin and hair. The SEM images indicated that the synthesized NPs were showing irregular particle structure and the size was ranging from 60–80 nm. TEM revealed that the shape and structure arrangement were crystalline in nature [49]. The biogenesis of rutile TiO_2_ NPs was produced utilizing an aqueous extract of Annona squamosa fruit peel. The green synthesis of rutile TiO_2_ NPs using agricultural waste is a simple, quick, ecologically sustainable, and less expensive process. In TEM, rutile TiO_2_ NPs have spherical forms and sizes ranging from 23 ± 2 nm. This study also includes SEM, UV, XRD, and EDS examinations. The powder particles have slight agglomeration, as evidenced by the SEM by the closed view of the spherical nanoparticles. The UV–Vis spectrophotometer revealed that TiO_2_ NPs resulted in a rapid, having a surface plasmon resonance at 284 nm. The XRD data revealed the relevant results to the JCPDS data (File No. 99-101-0954) [50]. In 2016, Madadi and Lotfabad synthesized TiO_2_ NPs by employing Acanthophyllum laxiusculum aqueous extract. This procedure of synthesizing nanomaterials is green or eco–friendly. The plant genus Acanthophyllum contains the richest sources of triterpene glycosides (saponins). TiO_2_ NPs are synthesized with the Sol-gel method. The sol-gel method is a common method for the synthesis of titanium dioxide NPs. In the sol-gel process, two steps occur: (1) hydrolysis of the Ti precursor in acidic or basic mediums; and (2) polycondensation of the hydrolyzed products [51]. This polycondensation can be prevented by using a surfactant such as natural surface-active compounds (NSAC) such as those that are utilized in this paper. This results in the formation of a collaborative framework, in which TiO_2_ NPs can be maintained. Scanning Electron Microscopy (SEM), TEM, UV, Energy Dispersive X–rays (EDAX), XRD, and were used to analyze TiO_2_ NPs. In this report, SEM images show particle sizes ranging from 20–25 nm, and TEM confirmed SEM data. The UV spectrum revealed an absorption band at 350 nm that corresponds to the optical band gap of 3.5 eV. Eventually, the FTIR confirmed the presence of TiO_2_ in the sample by peaks at 457, 470 cm^−1^, which revealed O—Ti—O bonding in anatase morphology. The relevant results of XRD data were found to be similar to JCPDS (File No. 21-1272) [35]. Furthermore, extract of Psidium guajava was used for the preparation of TiO_2_ NPs by Santhoshkumar et al. in 2014. The Synthesized TiO_2_ NPs were tested by disc diffusion method against human pathogenic bacteria. The XRD test revealed a dominant peak at 2θ = 27.57° and 41.37°, respectively, indicating the (110) crystallographic plane of anatase and (111) rutile form of TiO_2_ NPs. Peaks in the FTIR spectra of produced TiO_2_ NPs are 3410 cm^−1^ for C–H alkynes, 1578 cm^−1^, 1451 cm^−1^ for alkanes, and 1123 cm^−1^ for C–O absorption. FESEM was used to study the morphological characteristics of produced TiO_2_ NPs, which revealed a spherical shape and aggregates with an average size of 32.58 nm. Extracellular organic components are adsorbed on the surface of metallic nanoparticles, as evidenced by the presence of carbon, oxygen, magnesium, and chlorine, which were observed in EDX analysis [52]. In 2012, the TiO_2_ NPs were prepared using an aqueous extract of Jatropha curcas L by Hudlikar et al. XRD, Selected Area Electron Diffraction (SAED), TEM, EDAX, and FTIR spectroscopy were used to characterize the TiO_2_ NPs samples. The average size of TiO_2_ NPs was found to be in the range of 25–100 nm. XRD results were in agreement with JCPDS (File no. 84-1285) and TiO_2_ were nanocrystalline in nature and that was fair with TEM analysis. SAED confirmed the XRD concentric Scherrer planes of TiO_2_ NPs. The FTIR revealed the nature of the capping agent might be a peptide. This is due to the presence of C–H stretch, (N–H) stretch and carbonyl (–C–O–C–) or (–C–O–) stretch vibrations in the amide II and III bonding, before treatment of latex capped TiO_2_ NPs with 1% sodium dodecyl sulfate [53]. In 2016, Hunagund et al. employed the hydrothermal approach to synthesize TiO_2_ NPs with the support of a novel biogenic source, Piper betel leaf extract, and a chromogenic source, nitric acid, which acts as capping and reducing agents. Various characterization techniques were used on the synthesized TiO_2_ NPs, including UV–vis spectrophotometry, XRD, FTIR, TEM, which revealed that the NPs were spherical in shape with an average size of about 8–75 nm, and energy dispersive X-ray spectroscopy (EDS) for their optical, structural, morphological, and compositional investigations. The production of a rutile phase of TiO_2_ with a tetragonal crystal structure was clearly indicated by XRD patterns. The existence of certain sharp Bragg’s peaks were identified in XRD patterns, which could be related to the capping agent stabilizing the nanoparticles according to Hunagund et al. Intense Bragg’s reflection indicates high X-ray scattering centers in the crystalline phase, which could be attributable to capping agents [54]. Sundrarajan et al. (2017) investigated the synthesis of TiO_2_ NPs with the help of M. citrifolia leaves extract via the hydrothermal method. The TiO_2_ NPs had higher antibacterial activity against Gram-positive bacteria, suggesting their antimicrobial efficacy against pathogenic diseases, as per scientists. XRD, FTIR, UV–Vis diffuse reflectance (UV–Vis DRS), UV–Vis spectroscopy, Raman spectroscopy, and SEM with EDX techniques were used to evaluate TiO_2_ NPs. The peaks at 27.3° correspond to the (110) lattice plane of the tetragonal rutile TiO_2_ phase, and the average crystalline size of the NPs is 10 nm, according to the XRD study. The size of the NPs, between 15–19 nm, is readily visible in SEM imaging with EDAX spectra, which confirmed the formation of pure TiO_2_ nanopowder. Due to the quantum-confinement effect, green produced TiO_2_ nanoparticles have lower band gap energy than bulk pure TiO_2_ nanoparticles, which could have biological significance [55]. In 2013, Solanum trilobatum extract was used to make TiO_2_ NPs inhibit Pediculus humanus capitis, Hyalomma anatolicum, and Anopheles subpictus. XRD, FTIR, SEM, EDAX, and AFM were used to examine the green-produced TiO_2_ NPs [55]. Sankar et al. in 2014 prepared the TiO_2_ NPs by using aqueous leaf extract of Azadirachta indica under pH and temperature-dependent condition and the characterization were confirmed by UV–Vis spectroscopy and Fourier transform infrared spectrum. The interconnected spherical in shape TiO_2_ NPs with a mean particle size of 124 nm were revealed by SEM and dynamic light scattering (DLS) investigations and zeta potential of −24 mV [56]. In 2011, Velayutham et al. reported for the first time on the employment of aqueous extract of Catharanthus roseus to synthesize TiO_2_ NPs against Hippobosca maculata and Bovicola ovis. SEM analysis of the synthesized TiO_2_ NPs showed clustered and irregular shapes mostly aggregated and having the size of 25–110 nm [57]. From the kitchen waste collected, soaked Bengal gram beans (*Cicer arietinum* L.) were used for the synthesis of TiO_2_ NPs in this TiCl_4_ used as precursor. This is studied by Kashale et al. in 2016. Bio–TiO_2_ was systematically investigated by XRD, Raman spectroscopy, transmission electron microscopy (TEM), TGA, and BET surface area measurement system [48]. In 2013 Gautam Kumar Naik et al. informed the green synthesis of TiO_2_ NPs with the help of Cinnamomum Tamala leaves extract, which acts as the reductant. The structural and morphological properties of the nanocomposites were studied by X-ray diffraction, UV–visible diffuse reflectance, FT–IR, and transmission electron microscope [58]. The TiO_2_ NPs were prepared by Kandregula et al. using the fruit waste of Orange Peel extract as one of the precursors as it acts as a reducing agent and contains citric acid as the main source in its peel. The results were also shown by XRD, Particle Size Analyzer (PSA), Fourier Transform Infrared Spectrometer (FT–IR), and Thermo Gravimetric and Differential Thermal Analyzer (TG/DTA) [59]. With the use of Vigna radiata extract, Chatterjee et al. produced TiO_2_ NPs in 2016. Vigna radiata is a suitable source of reductant for the biosynthesis of these NPs. The findings revealed that oval-shaped TiO_2_ NPs could be biologically synthesized and that the particles were effective against both Gram-positive and Gram-negative bacteria. 1631.78 cm^−1^ and 1641.42 cm^−1^ in the FTIR spectrum suggested O–Ti–O bonding, while a peak at 3000 cm^−1^ occurred due to –OH stretching [60]. The TiO_2_ NPs manufactured from various plant species are shown in Table 1 below.

#### 2.4.1. Preparation of Plant Extract

The fresh leaves are thoroughly washed before being thinly sliced, then put in distilled water and kept boiling, after which the plant extract is filtered and ready to use, or the extract can be stored at low temperature for future use [24]. The thermal breakdown of phytochemicals occurs when leaves are heated. As phytochemicals (phenolic acids, alkaloids, proteins, including enzymes, and carbohydrates) are present in the plant extract, they are utilized in the reduction and stabilization stages [61].

#### 2.4.2. Titanium Dioxide (TiO_2_) NPs

TTIP (titanium tetra isopropoxide), TiCl_4_, TiO(OH)_2_ (metatitanic acid or titanyl hydroxide), and TiOSO_4_ (titanium oxysulphate) are some of the precursors that may be utilized to make TiO_2_ NPs (titanium oxysulphate) [62]. Depending on the application, the bulk TiO_2_ particles are dissolved in ethanol or distilled water. The obtained extract is then added into the mixture, drop by drop [63]. After that, the solution was stirred continuously at an appropriate temperature. The emergence of NPs causes a shift in the color of the solution [64].

Finally, the obtained NPs are filtered, distilled water washed, dried, and calcined. The synthesized NPs are stored in a furnace for calcination at temperatures ranging from 400–800 °C to remove excess organic groups [65]. Phytoconstituents in plants are supposed to fulfil at least one of the given functions, according to the classic green chemistry idea: metal salt reduction, hydrolysis of the Ti^4+^ precursor, solubilization, and polymerization of several intermediates [66].

**Table 1:** TiO_2_ NPs prepared by utilizing a variety of plants.

S/N	Plant Extract	Shape	Size (nm)	Ref.
**1.**	*Ageratina altissima*	Spherical	20–25	[35]
**2.**	*Azadirachta indica* leaves aqueous extract		124	[56]
**3.**	*Curcuma longa*		50–110	[67]
**4.**	Aqueous flower extract of *Calotropis gigantea*		160–220	[19]
**5.**	*Calotropis gigantea*		10	
**6.**	*Nyctanthes* leaves Extract		100–150	[68]
**7.**	Leaf aqueous extract of *Psidium guajava*	Spherical shape and clusters	32	[52]
**8.**	Flower aqueous extract of *Hibiscus* *rosasenansis*	Monodispersed and spherical	7	[69]
**9.**	Aqueous leaf extract of *Solanum* *trilobatum*	spherical and oval	70	[70]
**10.**	*Aloe vera* gel extract	Almost spherical	80–90	[71]
**11.**	0.3% aqueous extract of the latex of *Jatropha curcas* L.	spherical and uneven	25–100	[53]
**12.**	*Annona squamosa* peel extract	Polydispersed and spherical	23	[50]
**13.**	*Eclipta prostrata*	Polydispersed and spherical clusters	36–68	[72]
**14.**	Leaf extract of *Catharanthus roseus*	Clustered	5–110	[57]
**15.**	*Aloe vera*	Irregular	60	[73]
**16.**	*Aloe vera* leaves extract	Irregular structure	32	[49]
**17.**	Peelextractof *Citrus reticulata*	-	24	[59]

### 2.5. Microorganism–Based Synthesized of Titanium Dioxide NPs

**Figure 4:**
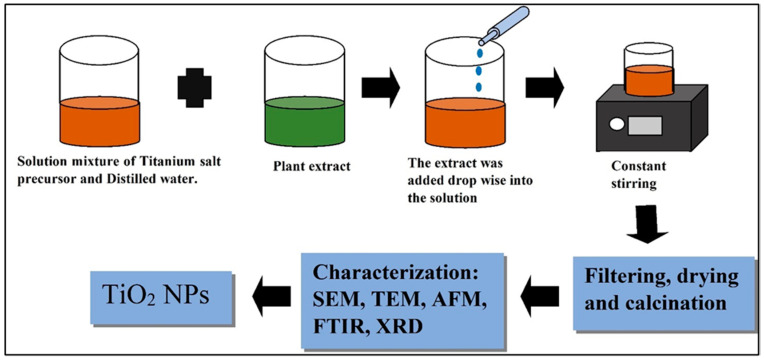
Schematic diagram of the synthesis process of TiO_2_ NPs using microorganisms.

**Figure 5:**
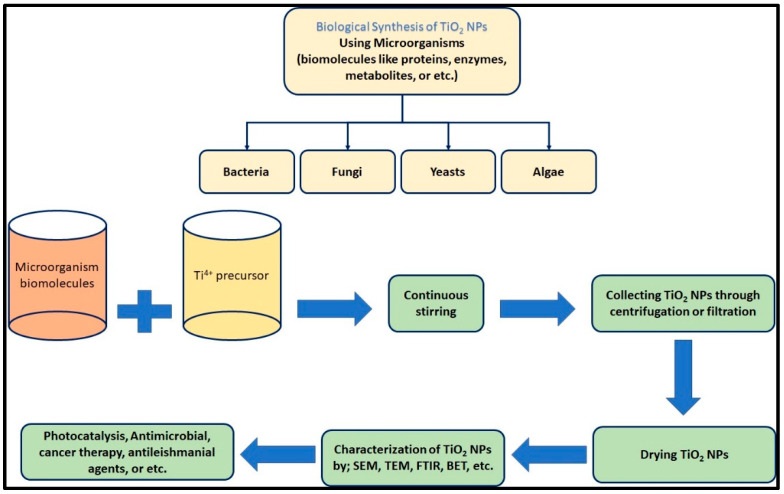
Green synthesis of TiO_2_ NPs.

In recent years, the biosynthesis of NPs using microorganisms has gained popularity as a more ecologically friendly alternative to chemical synthesis methods. These are inexpensive reagents with low toxicity and mild temperature and pressure requirements [74]. For various metal and metal oxide NPs, using microorganisms to generate NPs is a novel method [75]. The optical, chemical, photoelectrochemical, and electrical characteristics of NPs synthesized with microorganisms piqued researchers’ curiosity [76]. The formation of nanoscale materials by microbial cells is a promising method for the synthesis of metal nanoparticles. In environments with high metal concentrations, microbial synthesis can arise and develop. A variety of microorganisms are known to reduce metal ions into metal [77,78,79]. Figure 4 shows schematic diagram of the synthesis process of TiO_2_ NPs using microorganisms. In recent years, numerous forms and sizes of TiO_2_ NPs have been described. Bacterial extracts were utilized in the creation of green TiO_2_ NP production (green review). Bacterial metabolites, like plant extracts, play a key role in the bioreduction and stability of TiO_2_. *Aeromonas hydrophila* extract was used to manufacture 28–54 nm NPs that demonstrated effective inhibitory action against *Staphylococcus aureus* (33 mm inhibition zone) and *Staphylococcus pyogenes* (31 mm inhibition zone) [80]. The use of fungi to synthesize metallic NPs has gotten widespread interest, and they claim to have certain advantages over other bacterial production processes [81]. On the one hand, TiO_2_ NPs were manufactured utilizing the Lactobacillus bacterium during the combined action of oxidoreductase enzymes and glucose at moderate pH, while on the other hand, their possible pathogenicity and arduous bacterial manufacturing have minimal possibilities of being commercialized [82]. Mukherjee et al. revealed that the NPs developed had significant benefits, including scalability, facile extraction, high surface area, and economic feasibility. Through enzymatic reactions or metabolites, fungi can transform bulk salt into an atomic or ionic form [83]. The ability of extracts of Aspergillus flavus to reduce Ti ions precursors to TiO_2_ NPs was demonstrated in this study. These NPs demonstrated strong antibacterial action against *E. coli* [79]. Figure 5 shows a schematic diagram of the biological process for producing TiO_2_ NPs, as well as their characterization and applications. Saccharomyces cerevisa extract was also utilized to make TiO_2_ NPs, and SEM analysis revealed that the size of biosynthetic NPs was 12.6 nm. The existence of quinines and lipid reductases in the organisms was confirmed using FTIR analysis [82]. The surface properties and ionic strength of the culture medium too are important in the synthesis of TiO_2_ NPs. TiO_2_ NPs produced by fungi, like bacteria, have safety limits. Nonpathogenic strains, on the other hand, will eliminate the threat and may be commercially exploited [84]. Table 2 shows the synthesis of TiO_2_ NPs by various bacterial species.

## 3. Applications of Biogenic TiO_2_ NPs

The Green technique of NP generation has several applications in mechanical, electrical, and physical sciences, medicine, and engineering technology [94]. As compared to the biogenic TiO_2_ NPs, the NPs prepared by the microbial species showed a less significant number of practical applications. Green synthesis of NPs, on the other hand, shows a lot of potential when compared to physical and chemical techniques of production. The photocatalytic nanomaterials are commonly utilized to clean water and remove pollutants from the atmosphere [56,95,96,97]. Greenly produced TiO_2_ NPs offer a wide range of applications in electronics, energy generation devices, batteries, and sensors manufacturing [48,74]. The biosynthesized TiO_2_ NPs have also been used in biosciences, with photodynamic cancer therapy, antileishmanial agents, and antibacterial medicines among the applications [79,98,99]. The photocatalytic activity and antimicrobial efficacy of TiO_2_, as well as the most often used biomedical applications that apply mechanistic approaches, are discussed in the sections below.

### 3.1. Photocatalytic Activity of TiO_2_ NPs

The valence band has a complete energy level and is populated with electrons, whereas the conduction band has an unfilled energy level and is isolated from the valence band. An empty hole in the conduction band receives an electron from the valence band. The electrons in the valence band are transported to the conduction band to give TiO_2_ NPs their photocatalytic activity. As TiO_2_ is a semiconductor, photons of sufficient energy will cause it to produce electron-hole pairs [100]. The electrons in the valence band moved to the conduction band and filled the holes when UV light was absorbed on the TiO_2_ NPs. When conduction band-activated electrons and valence band holes react with water in the environment and oxygen; reactive oxygen species (ROS), hydroxyl radicals, and superoxide ions are formed [101,102]. In addition to hydroxyl and superoxide radicals, photocatalytic oxidation of nanoparticles, hydrogen peroxide, and singlet oxygen production occur. All of these radicals are known to be extremely reactive and can quickly destroy organic compounds when they come into contact with them [103,104]. Nowadays, household and industrial wastes contain a variety of hazardous and harmful substances, such as poisonous dyes and nitroarene compounds, which pollute the environment and cause water contamination. Hazardous dyes and other obnoxious substances have poor solubility and high stability, therefore justifying their tenacity and threat to aquatic life [105]. Freshly synthesized metallic NPs with a high catalytic capability and a specific structure were created. These metallic NPs also have a huge surface area, making them good heterogeneous catalysts [106]. The nanostructured catalysts also have the advantage of being easily recovered and recycled with the reaction mixture. The NPs’ toxicity, as well as their aggregation, are crucial aspects [107,108]. As a result of its high stability, low toxicity, optical properties, and photocatalytic potential, TiO_2_ NPs have largely been used in catalysis. Several studies claimed that green–mediated TiO_2_ NPs may be utilized to photo–catalytically reduce different dyes and compounds [56,97,109,110,111,112,113]. The sample was generated with 0.001 mol of TTIP precursor and yielded excellent results with lower particle sizes of 64.18 nm, indicating superior performance in photocatalytic activities [113]. The incubation composite displayed improved photocatalytic activity, while also degrading the rhodamine B dye and displaying maximum photocatalytic activity [112]. When green mediated NPs were compared to chemically synthesized TiO_2_ NPs for photocatalytic potential, green mediated NPs outperformed chemically prepared TiO_2_ NPs. The ability to reduce depends on phytochemicals found in plant species, the type of dye used, and the temperature [98]. The catalytic potential of doped TiO_2_ NPs with other metallic NPs was improved [114]. Table 3 shows the photocatalytic performance of TiO_2_ NPs that synthesized using different plant extracts [24,56,113,115,116,117,118,119].

### 3.2. Antimicrobial Potency of TiO_2_ NPs

**Figure 6:**
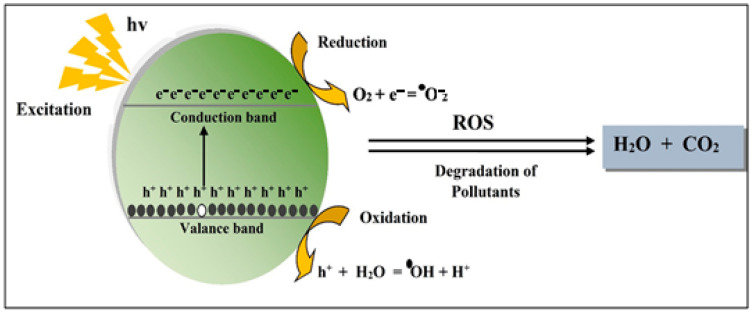
TiO_2_ NPs driven photocatalytic process in the presence of light.

Antimicrobial applications also make use of TiO_2_. In their investigation, Matsunaga et al. in 1988 found that TiO_2_ powder catalysts killed 99% of *E. coli* bacteria within 0.27 h when exposed to UV radiation (1800 µE m^−^^2^ s^−^^1^) [120]. This system is called a photo-sterilization system, which can be conducted in Figure 6. There are so many investigations that have been conducted to see the effect of TiO_2_ NPs catalysts on different bacteria. Maness et al. found that ROS formed on TiO_2_ surfaces, causing a lipid peroxidation reaction and the death of *E. coli* K–12 cells [121]. Numerous investigations have been conducted to examine how TiO_2_ NPs used for bactericidal purposes affect bacteria cells such as *E. coli*, *Pseudomonas aeruginosa*, *S. aureus*, *Enterococcus hirae*, *and Bacteroides fragilis* have been killed by the effects of TiO_2_ nanoparticles when exposed to UV light [122].

**Figure 7:**
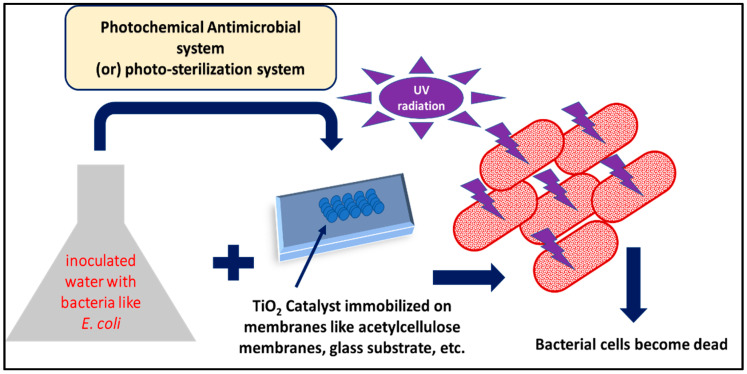
Simple experimental scheme for photochemical antimicrobial mechanism of TiO_2_ catalyst.

**Figure 8:**
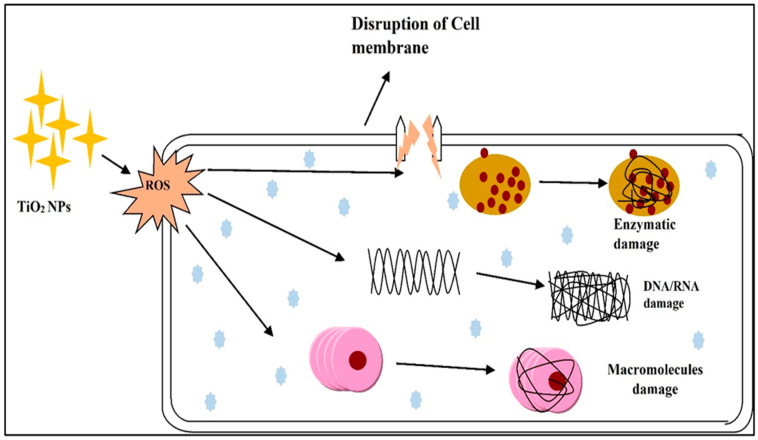
The impact of TiO_2_ NPs on microbes is depicted as a proposed pathway.

In the literature, biosynthesized TiO_2_ NPs were mediated and employed against several bacteria types [123]. Biosynthesized TiO_2_ NPs are ecologically friendly, have a high oxidizing potential, and are used in biomedicine. Biosynthesize TiO_2_ NPs were employed against a variety of bacteria, including strains, fungus, algae, viruses, and microbial toxins [124]. Table 4 reported the antimicrobial effect of TiO_2_ NPs against different bacteria. Figure 7 shows simple experimental scheme for photochemical antimicrobial mechanism of TiO_2_ catalyst. The impact of TiO_2_ NPs on microbes is depicted in Figure 8 as a proposed pathway. When TiO_2_ NPs come into contact with microbial cells, they form reactive oxygen species (ROS) [80]. ROS acted to reduce adhesion by killing bacteria by disrupting cell wall integrity, stopping respiratory cytosolic enzymes from changing macromolecule structures, and having strong effects on cellular integrity and gene expression. Phosphate uptake and cellular communication are also inhibited [80,125]. In comparison to both green produced and chemically generated TiO_2_ NPs, bio-synthesized NPs showed greater antibacterial activity. The capping agents obtained from plant extracts are credited with their excellent antibacterial properties [69]. The antibacterial action of NPs is influenced by their structure, membrane biology, and bacteria species. Green TiO_2_ NPs are employed to slow both Gram-positive and Gram-negative bacteria, albeit Gram-positive bacteria are less reactive than Gram-negative bacteria due to their structural complexity [19]. If bio–mediated TiO_2_ NPs are irradiated with UV and fluorescent light, their antibacterial activity can be improved [50,80]. When green-produced TiO_2_ NPs were introduced to Leishmanial cells, they demonstrated enhanced antileishmanial activity as well as decreased cell viability, slow growth, and DNA fragmentation [125]. TiO_2_ NPs surpassed typical antibiotic discs in terms of antibacterial activity [52].

## 4. Future Challenges

Synthetic methods involving fungus, bacteria, and other organisms are complex due to strain separation and difficulties in growth. These processes are also difficult owing to the need to maintain the culture media, as well as the physical and chemical conditions. Plants are selected primarily since they are simple to extract and plentiful. This approach might be used to regulate the size, shape, and crystalline structure by adjusting the experimental parameters. Despite this, only a few plants are exploited in the phyto-synthesis of TiO_2_ NPs, and additional study is urgently required in this field. These phyto-synthesized nanoparticles may be used safely not just in biomedical activities, but also in all other potential applications as they are similarly compatible with chemically produced nanoparticles. As previously stated, the crucial aspects of NP are determined by their size and shape. As a result, future difficulties will include figuring out how to leverage similar biological techniques to make various forms including triangular, cuboidal, truncated, ellipsoidal, pyramidal, decahedral, and oval. Scaling up NP production from the lab to the commercial-scale is a tough process with many challenges and unknowns. There are two more obstacles to overcome. All across the production process, cost, dependability, waste, energy consumption, recycling possibilities, material safety, and hazard level should all be addressed. Furthermore, the properties of nanomaterials may change as they scale up. The amount of control may be diminished when dealing with large volumes.

## 5. Summary

The recent research effort in the topic of biogenic synthesis of TiO_2_ NPs using plants and microbes has been discussed in this review. It also delves further into the mechanism of TiO_2_ NPs’ phyto-synthesis. Despite metallic nanoparticles being formed through different physicochemical processes, their cytotoxicity, high cost, and time consuming production have prompted scientists to propose new nanostructures design methods. The formation of titanium dioxide nanoparticles from various biological sources (plants, microorganisms, and related bioproducts) has been discussed. Furthermore, the mechanism of their uptake, translocation, and accumulation in plants are explored. The potential impact of TiO_2_ has also reported. The green synthesis is being promoted due to a number of significant advantages associated with this technique. This approach might be used to regulate the size, shape, and crystalline structure by adjusting the experimental parameters. Despite this, only a few plants are exploited in the phyto-synthesis of TiO_2_ NPs, and additional study is urgently required in this field. These phyto-synthesized nanoparticles may be used safely not just in biomedical activities, but also in all other potential applications as they are similarly compatible with chemically produced nanoparticles. Apart from biomedical and environmental remediation applications, further scientific research should be devoted to finding practical uses of phyto-synthesized NPs in other fields. To summarize, green technology via biosynthesis, as discussed in the article, yields outstanding insights that may encourage foster researchers and beginners to proceed and expand their investigation of nature’s potential, as well as the development of effective and sustainable methodologies for nanoparticle synthesis with desirable characteristics, which can be utilized in a variety of disciplines.

## Figures and Tables

**Table 2:** TiO_2_ NPs produced by several bacterial communities.

S/N	Bacterial Species	Shape	Size (nm)	Ref.
**1.**	*Aeromonas hydrophila*	Spherical	40–50	[80]
**2.**	*Bacillus amyloliquefaciens*		22.1–97.2	[78]
**3.**	*Bacillus subtilis*		30–40	[85]
**4.**	*Bacillus subtilis*	Spherical	66–77	[86]
**5.**	*Bacillus subtilis*		10–30	[75]
**6.**	*Lactobacillus*		8–35	[82]
**7.**	*Lactobacillus*		40–60	[87]
**8.**	*Planomicrobium*		100	[88]
**9.**	*Aspergillus niger*		73.58	[89]
**10.**	*Fusarium oxysporum*		10	[90]
**11**	*Aspergillus flavus*		62–74	[79]
**12.**	*Bacillus mycoides*	Polydisperse	40–60	[74]
**13.**	*Fusarium oxysporum*	Quasi–spherical	9.8	[91]
**14.**	*Aspergillus tubingensis*	-	<100	[92]
**15.**	*Aspergillus niger*, *Rhizoctonia bataticola*, *Aspergillus fumigatus*, *and Aspergillus oryzae.*	-	-	[93]

**Table 3:** Photocatalytic effect of Titanium dioxide nanoparticles using different plant extracts.

S/N	Dye	Concentration of Dye	Catalyst Dosage	Exposure Time	Percentage Removal	Ref.
**1.**	methylene blue (MB) dye	6, 10, 20, 40 ppm	0.1–0.4g	6 mg. L^−1^ of MB in 45 min	13.3%	[113]
**2.**	methylene blue, alizarin red, crystal violet, and methyl orange	10 mg/L	50 mL	6 h	86.79%, 76.32%, 77.59% and 69.06%	[115]
**3.**	methyl orange	-	1 g/dm^3^	150 min	94%	[24]
**4.**	RO–4 dye	-	15 mg, 20 mg, 25 mg and 30 mg	180 min at 3.5 pH	91.19%	[116]
**5.**	methyl red	10 ppm and 20 ppm	1 g/L	60 min	89% and 83%	[117]
**6.**	methyl red	50 mL	10 mg	120 min	-	[56]
**7.**	Methyl Blue	200 mL	10 mg	75 min	-	[118]
**8.**	indigo blue dye	1 ppm at pH 6.0	-	150 min	75%	[119]

**Table 4:** Antimicrobial effect of Titanium dioxide NPs using different bacteria.

S/N	Catalyst	Dosage	Species Name	Zone of Inhibition	Ref.
**1.**	TiO_2_	25 µg mL^−1^, 20 µg mL^−1^, 30 µg mL^−1^, 10 µg mL^−1^, 10 µg mL^−1^, 15 µgmL^−1^	*A. hydrophila*, *E. coli*, *P. aeruginosa*, *S. pyogenes*, *S. aureus*, *E. faecalis*	23 mm, 26 mm, 25 mm, 31 mm, 33 mm, 16 mm	[80]
**2.**	TiO_2_	20 μg/mL, 40 μg/mL	*E. coli*		[74]
**3.**	TiO_2_	20 μg/mL	*S. aureus* *and E. coli*	25 mm, 23 mm	[52]
**4.**	TF–TiO_2_	20 μL of 10 mg/mL	*S. aureus*, *S. faecalis*, *E. coli*, *E. faecalis*, *Y. enterocolitica*	11.2 mm, 11.6 mm, 10.8 mm, 11.4 mm, 10.6 mm	[94]
**5.**	*A. flavus* synthesized TiO_2_	40 µg mL^−1^, 40 µg mL^−1^, 80 µg mL^−1^, 70 µg mL^−1^, 75 µg mL^−1^	*S. aureus*, *E. coli*, *P. aeruginosa*, *K. pneumonia*, *B. subtilis*	25 mm, 35 mm, 27 mm, 18 mm, 22 mm	[79]

## Data Availability

Not applicable.
